# Maternal perspective on requesting elective caesarean birth

**DOI:** 10.12669/pjms.42.1.10534

**Published:** 2026-01

**Authors:** Nayyer Sultana, Maha Shafqat

**Affiliations:** 1 Nayyer Sultana, MBBS, FCPS, CHPE Professor, Department of Obstetrics and Gynaecology, Central Park Medical College, Lahore Pakistan; 2 Maha Shafqat, MBBS Postgraduate Resident, Department of Obstetrics and Gynaecology, Central Park Medical College, Lahore Pakistan

**Keywords:** Elective caesarean section, Planned caesarean section, Maternal wish, Primigravida

## Abstract

**Objective:**

This study was conducted to explore the underlying factors for requesting elective caesarean section without an obstetric indication in primigravida.

**Methodology:**

A qualitative case study was conducted in Obstetrics & Gynaecology department at Central Park Hospital from September 2023 to February 2024. The study participants were primigravidas who presented after 37 weeks of gestation and requested for caesarean section without any medical indication. Semi-structured interviews were taken from fifteen participants for an in-depth analysis of the causes of elective caesarean section. Thematic analysis was done using NVivo 14.

**Results:**

The analysis of the reasons given by women to seek caesarean section led to the identification of four main themes: psychological fear, negative birth experiences, behavioral control, and maternal firmness. Traumatic birth experiences of near relatives and friends emerged as a main reason that resulted in fear of childbirth. Psychological fear related to labour pains and vaginal examination were yet another reason. Behaviour control expressed as distress after witnessing another woman’s labour, ashamed of exposure and lithotomy positioning were described by few of participants. Most respondents reported more than one reason for requesting caesarean section.

**Conclusion:**

We found that CDMR in primigravidas is multifactorial and had personal, behavioural and social contexts. Listening to horrific birth stories from surroundings was most pronounced factor that created a negative impression of vaginal delivery on first-time mothers and generated self-perceived harm which led to multi-faceted fear regarding birth process.

## INTRODUCTION

World Health Organization (WHO) report suggests that an estimated 10-15% caesarean section rates are acceptable and above it is unwarranted.^1^ Worldwide trends of caesarean section have been seen to rise from 7% in 1990 to 21% in 2021.^2^ According to Pakistan demographic and health Survey 2017-2018, the rate of caesarean section was 19.6% whereas it was 3.2% in 1990-1991.^3^ Maternal request for caesarean delivery is one of the contributing factors to this rising trend. Caesarean delivery on maternal request (CDMR) has been reported in literature worldwide with varied incidence ranging from 0.2-42%.^4^ In Pakistan it was reported to be 6.6%.^5^

The ethical justification for performing CDMR is complex and requires balancing maternal autonomy, clinical safety, and provider responsibility.^6^,^7^ Respect for a woman’s autonomy is a fundamental principle in medical ethics. If a woman is fully informed about the risks and benefits of both mode of deliveries and is making an informed choice based on her preferences, values, and circumstances, her autonomy should be respected.^8^ Different healthcare professionals and societies may have varying opinions on this matter.

American College of Obstetrics & Gynaecology (ACOG) guidelines favour CDMR provided woman receives adequate information and counselling whereas Canadian guidelines recommend caesarean section only on obstetric indications.^9^,^10^ Ethical perspectives may differ regarding the balance between a woman’s autonomy and the responsibility of healthcare professionals to prioritize the well-being of both the mother and the baby. Some argue that if there is no medical necessity, healthcare providers should carefully consider the potential risks of unnecessary surgery. While some practitioners accept CDMR as it is thought to be a safer mode of delivery and to prevent them from litigation.^11^ Adherence to clinical guidelines will prevent obstetrician bias and unnecessary caesarean section in nulliparous women.^12^

Mothers who desire an elective caesarean section may do so for a variety of reasons, such as fear of labour pains, worry for their child’s safety, prior negative birth experiences, convenience of scheduled delivery, and positive perceptions of caesarean section for its painless and swift process. It is also noted that the reasons and rate of CDMR varies country by country and between regions of same country.^13^ Although many studies have investigated the prevalence of caesarean delivery on maternal request, only few qualitative studies have explored the deep-lying psychosocial and cultural factors driving this preference in Pakistan. Quantitative methods cannot capture the complex motivational influences and perceptions that underlie women’s decisions regarding mode of delivery. A qualitative approach was thus used to probe into the personal experiences, beliefs, and contextual influences that contributed to the request for a planned caesarean section The data on in-depth analysis of reasons behind CDMR in our context is lacking. The objective of this study was to explore the reasons for requesting planned caesarean section without an obstetric indication in primigravida.

## METHODOLOGY

This was a qualitative case study conducted in the department of Obstetrics & Gynaecology at Central Park Hospital from September 2023 to February 2024. Qualitative data was collected for an in-depth analysis of the causes of caesarean section without medical indication in first time mothers.

### Ethical approval:

it was taken from institutional review board & IRB number was CPMC/IRB-No/1405. Date August 09, 2023.

All women in their first pregnancy who requested for a caesarean section and elective caesarean delivery was planned were included in the study. While primigravidas who had caesarean section for obstetric indications and women with any parity other than primigravida were excluded. Written consent was taken from all study participants. The interviews were conducted either in the antenatal ward before scheduled caesarean section or in the postnatal ward before discharge. An interview guide was made and semi-structured interviews were taken from all participants in their native language at a quiet place with complete privacy, each interview lasting 30-40 minutes. Semi-structured interviews were used to explore this sensitive topic in depth. Interviews frequently adopted a narrative format using open ended questions and began with the opening query, “Would you like to tell me your story?” Discuss why you desire a planned caesarean section?”, and if deemed necessary, the author continued with inquiries and follow-up questions from the interview guide. All interviews were tape recorded after prior consent from study participants. Later, all interviews were transcribed in English language. During data collection, transcripts were sequentially read to modify the interview guide, to assess saturation, and to identify key topics for additional study. After fifteen interviews further interviews were stopped.

**Fig.1:**
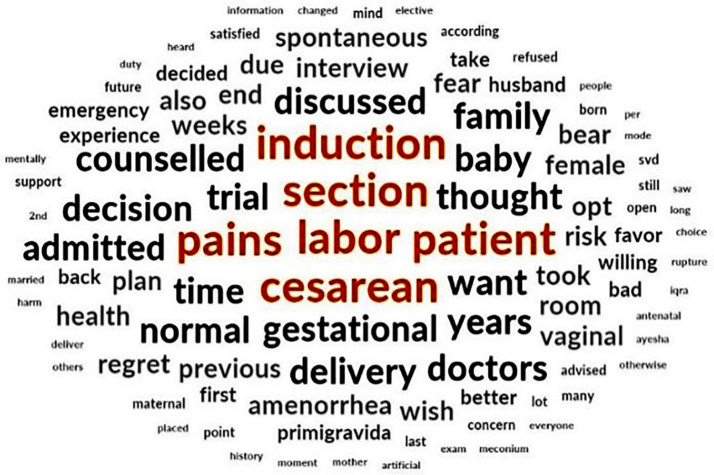
Word query for theme search.

**Fig.2:**
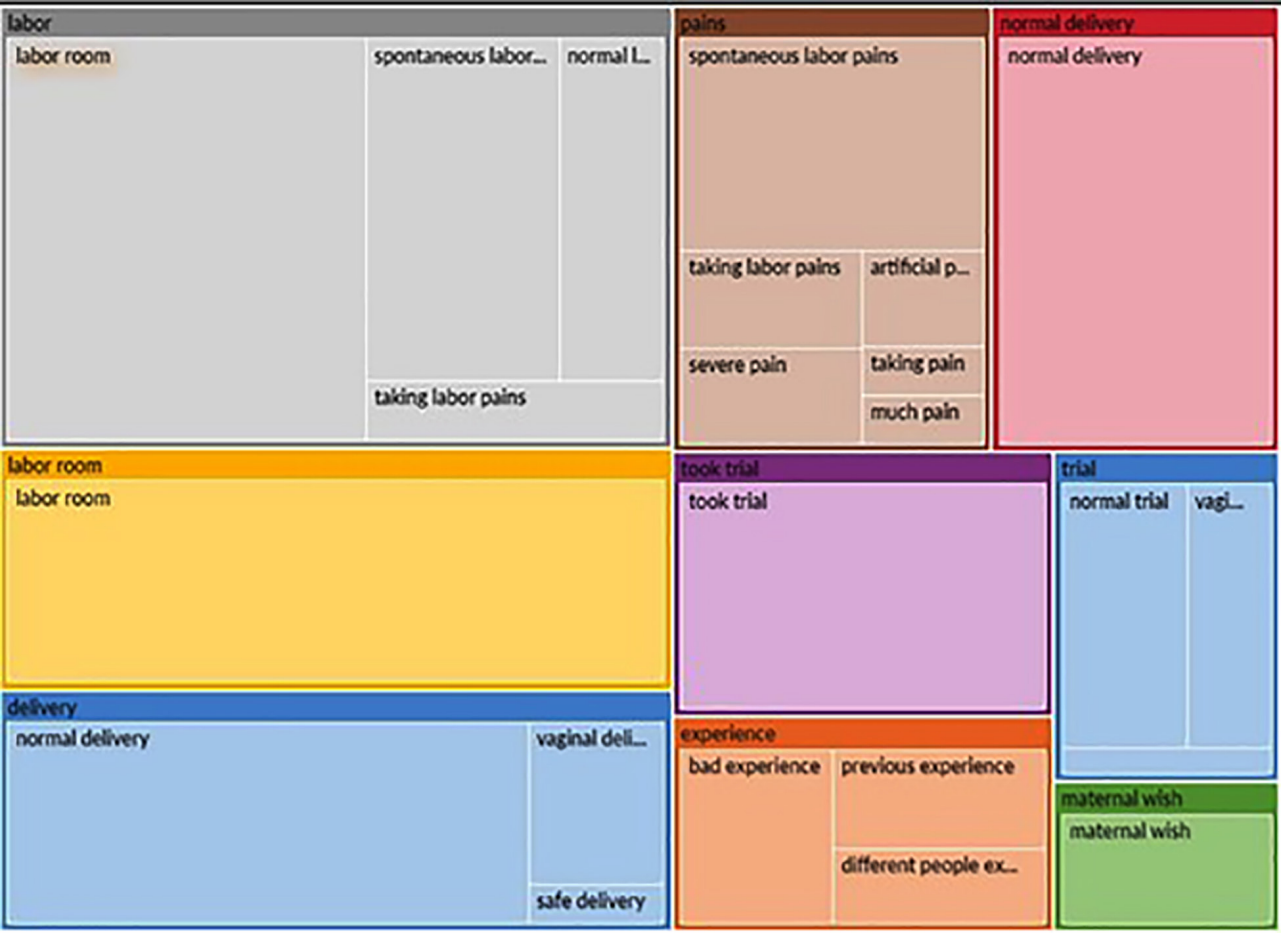
Hierarchy diagram of auto-codes.

Data were analyzed using an inductive thematic analysis. Transcripts were imported into NVivo 14 for data management while all codes were generated manually by the authors through line-by-line reading. NVivo tools-for example, word-frequency queries and auto-coding suggestions-facilitated the identification of repeated ideas shown in Fig.1 & Fig.2. However, the coding framework and themes were created by the researchers. Through constant comparison, codes were refined, and related codes were combined into categories to inform the final themes.

Credibility was enhanced through independent coding, consensus discussions, and prolonged immersion in the data. Dependability was ensured by an audit trail detailing coding decisions and theme development, as well as through an iterative process of updating the interview guide. Confirmability was promoted through reflexive note-taking and through analytic triangulation by two researchers. Finally, four themes emerged. To enhance the study’s rigor and strengthen its overall validity, an independent reviewer examined the entire project. This external audit offered valuable feedback on elements including transcription precision, the linkage between data and research questions, and the analytical progression from raw data to final interpretation.

## RESULTS

The age range of study participants was between 23-29 years. Two participants had education up to matric, three till intermediate, nine had education up to graduation and only one participant had done Masters. About half of the participants belonged to upper middle class and half belonged to lower middle class.

### Main categories:

The analysis of the reasons given by women to seek caesarean section led to the identification of four main themes and further sub-themes shown in Table-I. Traumatic birth experiences of near relatives and friends emerged as a main reason, and resulted in a preference for a planned caesarean section as a way of avoiding these situations. Psychological fear related to labour pains and vaginal examination were yet another reason for caesarean section. Behaviour control expressed as distress after witnessing another woman’s labour, ashamed of exposure and lithotomy positioning were described by few of participants. The women were also found to be very firm in their decision as they also received their family support.

**Table-I:** Themes & Sub-themes.

Themes	Sub-themes
*Influence of negative birth experiences in the surrounding*	Harm to baby
	Instrumental delivery
	Refusal for induction of labour
	Labour trial would fail
*Psychological fear*	Fear of labour pains Fear of vaginal examination
*Behaviour control*	Distress after witnessing another woman’s labour
	Ashamed of exposure & lithotomy position
	Sexual dysfunction
*Maternal firmity regarding birth decision*	Women’s autonomy
	Family support
	Safety of the procedure
	Small family size

### Influence of negative birth experiences in the surrounding:

It was discovered that by listening to the birth experiences of close friends and relatives had a great impact on first time mothers’ decision regarding mode of delivery.

### Harm to baby:

The fear of losing a baby during childbirth is a profound and deeply distressing concern that was coded by many women. These women witnessed their near ones in deep mental trauma after losing their baby and decided not to repeat the same mistake themselves.

“My cousin’s baby could not survive while taking labour trial”. (R-8):

### Instrumental delivery:

For some, there was a phobia of instrumental delivery generated because of traumatic operative delivery of some close friend or relative that made them think of a nightmare.

*“I don’t want to go through the same horrific childbirth”* *(R-1)*

### Refusal for Induction of labour (IOL):

Some of the study participants had prolonged pregnancy and offered induction of labour (IOL) with prostaglandins. The women perceptions about IOL involved mixed opinions such as prolonged labour, increased intensity of pain and need of instrumental delivery.

*“I heard induction of labour is harmful for both mother and the baby. I could not risk two lives just for the sake of vaginal birth.” (R-12)*

Labour trial would fail: Most of the study participants believed that their labour trial would fail.

“she went through two birthing processes”:

Details of other excerpts related to influence of negative birth experiences in surroundings, that are not described here, are given in annexure 1.

**Annexure 1:** Sub-themes & Excerpts related to influence of negative birth experience in the surrounding.

Theme	Sub-themes	Excerpts
influence of negative birth experience in sur-rounding	Harm to baby	*“My friend was undergoing a labour trial. Her labour lasted several hours, and at the very last second, her baby passed meconium within her womb. The infant’s heart rate began to decrease, and at delivery, the baby did not have a chance of survival.* *“(R-6)*
		*“I am also concerned for my baby’s safety. I think planned cesarean is a better choice than artificial labour.” (R1)*
		*“My top priority is for my child to be delivered safely. I don’t want to put my child in danger in order to receive a medal for a vaginal birth.” (R-10)*
		*“I am also concerned for my baby’s safety as prolonged labour could harm my child.” (R2)*
		*“During her labour trial, my friend lost her baby. Her labour lasted a long time, and she was so tired that she was unable to push while the baby’s heartbeat was decreasing. Her baby did not survive after going through all of that suffering. The birth experience should be one that you can cherish when you recall rather a horrible night mare.” (R7)*
		*“As I conceived long time after marriage, this baby is very precious to us. we don’t want to take any risk. Please do my cesarean section.” (R9)*
	Refusal for induction of labour	*“I would have no objection for a vaginal birth if my labour pains were spontaneous. As my doctor advised me for induction of labour, I would prefer a cesarean delivery instead. (R12)*
		*I heard induction of labour is harmful for both mother and the baby. I could not risk two lives just for the sake of vaginal birth.” (R12)*
		*“My delivery is planned through induction of labour as I have crossed my due date. I heard labour induction leads to prolonged duration of labour. I don’t think I could withstand the lengthy duration of labour. I don’t have enough strength.” (R2)*
	Labour trial would fail	*“I can’t handle the stress of uncertainty. Can you assure me of a vaginal delivery? What if I start having labour pains and you urge me to have an emergency caesarean section at the last minute?” (R10)*
		*“I’m worried that at any time my labour could become stuck and I’d need to have a caesarean section. If I wouldn’t be giving birth vaginally, then what use would it make to take labour pains? I believe I should have a caesarean section straightaway.” (R5)*
		*“”Can you give me the surety of safe normal vaginal delivery? What if my labour would prolong to extended hours and normal delivery would be impossible and we have to switch to an emergency cesarean? I don’t want to experience two painful procedures. Please let it be only one.” (R-2)*

### Psychological fears:

### Fear of labor pains:

As none of them ever experienced this event before but they related this pain to their past experiences of different types of pains like period pain or pain of intravenous injection.

*“I don’t think I’m capable of handling labour pains. Even in period days I used to take off from my studies. The level of anguish caused me to start throwing up. I don’t have the patience for childbirth pains.”* *(R-14)*

### Fear of vaginal examination:

The women’s experiences were a terrible combination of suffering, feeling ashamed, and a worst thing ever.

“I never had a vaginal examination. However, I believe that I could never have a pelvic examination as it could be the most excruciating and embarrassing thing ever.” (R-15)

### Behavioral control:

### Distress after witnessing another woman’s labour:

Visual experience changed decision regarding mode of delivery for some women.

“I was prepared for a vaginal birth, but while I was in the labour room, I witnessed women in excruciating pain. Something about this visual impaction has made me restless.” (R10)

### Ashamed of exposure & lithotomy position:

One of the women requested a caesarean section just because lithotomy positioning was unacceptable for her.

*“In order to make myself prepared for normal vaginal delivery, in the past week I watched videos of normal delivery. I saw the woman in lithotomy position and exposed in front of so many people including obstetrician, a junior doctor and two or three paramedical staff. It’s hard for me to comprehend this truth when I see myself in the same circumstance.” (R-11)*

### Sexual dysfunction:

One of the participants believed normal delivery would lead to laxity of perineum and vagina that could lead to disinterest of her husband during intercourse due to lack of sexual pleasure.

“I heard that normal deliveries lead to loosening of vagina and widening of perineum. This deformity of vagina and perineum may lead to loss of sexual pleasure.” (R-14)

Maternal firmity regarding birth decision:

*“Although, I had a long debate with my obstetrician, but I am not convinced to get a trial of labour**.** I think I do not require any further guidance as my decision is firm.” (R-3)*

The family support also encouraged first-time mothers to gain confidence in their decision.

“I have discussed my concerns with my family, and they supported my opinion.” (R-1)

Some women believe that caesarean section is a safe procedure because they did not witness any of the complications related to the procedure.

“Moreover, I did not see any of the complication of caesarean section in my friends and family.” (R-14)

One mother used the argument of having a small family as justification for a caesarean section.

“My doctor explained me all complications of caesarean section. However, I don’t desire a large family. We had only two children in mind already. Therefore, I didn’t care that I had two caesarean deliveries.” (R-4)

## DISCUSSION

Our study brings new insights into the reasons for requesting elective caesarean among primigravida in our local context; an area where evidence remains sparse. Unlike previous research, which has focused mostly on multiparous or general populations, ours demonstrates that first-time mothers can form strong early preferences for a caesarean birth despite never having been in labour.

The frequent reasons identified were fear of harm to unborn baby, uncertainty of birthing process related to instrumental delivery, perception of failed trial of labour necessitating emergency caesarean section and primary fear of labour pains. Almost all women were very certain and firm regarding their request for caesarean section. Additionally, our analysis revealed other less frequently reported reasons, such as refusal for labour induction, fear of repeated vaginal examinations and difficulties with behavior control related to exposure and lithotomy positioning. These multitude of reasons are aligned with the existing literature.^14^-^16^ Top of Form

Since our study participants lacked prior birthing experience, we observed that these women had a self-perceived sense of risk, either towards themselves, the birthing process, or the unborn baby. This perception of risk stemmed from either hearing about or witnessing traumatic birth experiences in their environment. Influence of negative birth experiences in near relatives has a significant role in modifying personal beliefs and inclination towards caesarean section.^17^ In literature this phenomenon is described as prospective fear that includes both social and personal aspects. The social aspect described the fear of childbirth generated in primigravidas as a result of listening to horrific birth stories and personal aspect was explained as primary fear of childbirth.^18^ When a mother develops fear of childbirth, she demonstrates negative thoughts regarding outcome of labour, harm to baby, poor control on herself and frequently asks for caesarean section.^19^

Yet another interesting reason for CDMR revealed was refusal for induction of labour (IOL). Women believed that IOL would be detrimental to both themselves and their unborn child. Escape from IOL as a request for a caesarean section is less reported in the literature. Masciullo et al. did a quantitative analysis of CDMR and found refusal for medical IOL as one of the reasons but that analysis did not provide insights to this request.^14^ One respondent expressed her concern of being exposed in front of a large crowd and she did not want herself to be presented as a teaching case. Concerns about exposure during teaching examinations and discomfort with lithotomy positioning also emerged as distinct contributors, underscoring the need for respectful maternity care practices. Similar concerns are only minimally described in the literature .^20^

It was discovered that women who chose to have a caesarean section were quite adamant about their choice and did not need any further information. Majority of women were graduates or had higher education. Mothers with more education have been shown to assert that choosing the mode of birth is their inherent right. In Poland this trend was 25% in 2010 and this figure grown to 35% in 2020.^21^ It has also been observed that post-traumatic stress disorder develops in women who have a fear of giving birth and whose request for a caesarean section is denied.^22^

A lady who requests elective caesarean birth should be assessed for fear of childbirth (FOC) as it could be detrimental to health in 1.6% of cases. Antenatal training classes has a significant role in reducing FOC.^23^ Maternity services should implement counseling programs especially for first-time mothers, to address misconceptions, fears, and anxieties surrounding childbirth. Health care providers should ensure that women’s privacy and dignity are respected during childbirth by addressing concerns related to exposure during labour and delivery. Since there isn’t yet full consensus on the CDMR, if after adequate counseling, a woman still insists for elective caesarean, her request should be respected to protect her from traumatic psychological event. Future studies should include diverse settings, particularly public-sector hospitals where patient demographics differ significantly. Research exploring the effectiveness of antenatal counseling, fear-reduction interventions, and privacy-enhancing measures may further inform strategies to reduce unnecessary caesarean requests.

### Strength & Limitation:

A key strength of this study is the analytical rigor achieved through dual independent analyses by researchers with complementary professional backgrounds—one with expertise in qualitative methodology and the other with clinical experience in obstetrics. This triangulation allowed the data to be interpreted from different disciplinary perspectives, enhancing the credibility and depth of the findings. Since this study was conducted in a private-sector hospital that primarily serves women from relatively advantaged socioeconomic backgrounds, the findings may not fully reflect the experiences of women from other social strata. To enhance the transferability of future research, similar studies should be conducted in public-sector hospitals as well, where patients come from a broader range of socioeconomic and demographic circumstances.

## CONCLUSION

We found that CDMR in primigravidas is multifactorial and had personal, behavioural and social contexts. Exposure to negative birth narratives from close social circles was most pronounced factor that created a negative impression of vaginal delivery on first-time mothers and generated self-perceived harm which led to multi-faceted fear regarding birth process. Interventions should target the decision-making period when women begin to consider caesarean delivery, to address underlying fears and misconceptions. Doing this should also include educating mothers on the birth decision-making process to reduce the level of fear mothers have around the process of childbirth.
